# Road-Aware Trajectory Prediction for Autonomous Driving on Highways

**DOI:** 10.3390/s20174703

**Published:** 2020-08-20

**Authors:** Yookhyun Yoon, Taeyeon Kim, Ho Lee, Jahnghyon Park

**Affiliations:** Department of Automotive Engineering, Hanyang University, Seoul 04763, Korea; yyh6920@hanyang.ac.kr (Y.Y.); raspbe34@naver.com (T.K.); ho3021@gmail.com (H.L.)

**Keywords:** trajectory prediction, high-definition maps, highway driving, curvilinear coordinates, lane assignment

## Abstract

For driving safely and comfortably, the long-term trajectory prediction of surrounding vehicles is essential for autonomous vehicles. For handling the uncertain nature of trajectory prediction, deep-learning-based approaches have been proposed previously. An on-road vehicle must obey road geometry, i.e., it should run within the constraint of the road shape. Herein, we present a novel road-aware trajectory prediction method which leverages the use of high-definition maps with a deep learning network. We developed a data-efficient learning framework for the trajectory prediction network in the curvilinear coordinate system of the road and a lane assignment for the surrounding vehicles. Then, we proposed a novel output-constrained sequence-to-sequence trajectory prediction network to incorporate the structural constraints of the road. Our method uses these structural constraints as prior knowledge for the prediction network. It is not only used as an input to the trajectory prediction network, but is also included in the constrained loss function of the maneuver recognition network. Accordingly, the proposed method can predict a feasible and realistic intention of the driver and trajectory. Our method has been evaluated using a real traffic dataset, and the results thus obtained show that it is data-efficient and can predict reasonable trajectories at merging sections.

## 1. Introduction

Driving situation awareness is a fundamental requirement for an intelligent vehicle, since high-level decision making, trajectory planning, and tracking control are based on this information [1,2]. In particular, trajectory prediction of surrounding vehicles is one of the key elements for understanding driving situations [3]. Furthermore, long-term trajectory prediction has certain advantages; for example, a vehicle equipped with trajectory prediction can not only avoid an accident, but also generate evenly distributed control input sequences, such as a jerk-minimizing acceleration, by reacting in advance. However, this is a challenging task since an autonomous vehicle cannot directly measure the intention of a driver using sensors [4] and each driver has different driving characteristics. Thus, an accurate prediction algorithm is required to take this uncertain nature of the future into consideration.

The commonly used classical method for predicting the state of a vehicle was the Kalman filter (KF) [5,6]. KF-based approaches still exhibit good performance for short-term prediction. However, the longer the prediction horizon, the lower is its accuracy, since KF does not account for the uncertain nature of the driver’s maneuvering. To tackle this limitation, many studies have adopted learning-based approaches, such as recurrent neural network (RNN) variants [7,8,9] and a combinatorial model of a variational autoencoder (VAE) and an RNN encoder–decoder structure, to improve the prediction accuracy [10,11,12] of the employed network.

**Figure 1:**
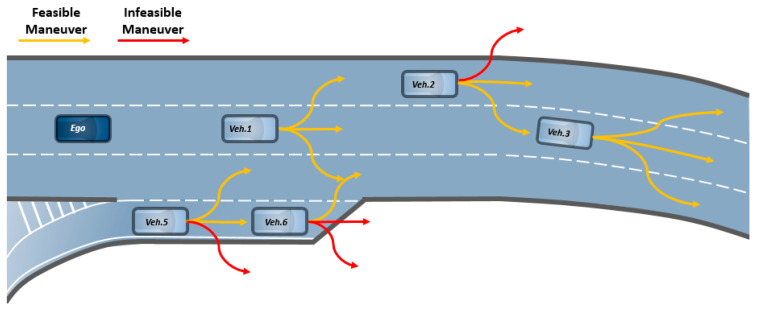
Schematic showing the concept of road-aware trajectory prediction: vehicles should run along roadway while obeying the structural constraints of the road.

On the contrary, an on-road vehicle motion is constrained to the road shape. In other words, the vehicle should run along the roadway while obeying the structural constraint of the road (see Figure 1). Due to this characteristic, high-definition maps (HD maps), which contain detailed road information, have recently been used as a useful element in various autonomous driving applications such as vehicle localization [13] and on-road vehicle tracking [14,15]. Despite a wide variety of benefits, the use of road information in the field of trajectory prediction is very limited. Therefore, we propose a novel methodology for maximizing the usage of HD maps for trajectory prediction on highways.

The contributions of this study are as follows:

- We have developed a practical data-efficient method for trajectory prediction on highways using the curvilinear coordinate system and lane assignment. It is inevitable to collect a real dataset by driving along a roadway. However, it is also impossible to collect all the necessary data that can cover the whole range of the sensor. To mitigate this practical problem, we have proposed a data-efficient learning method to make the dataset compact. In addition, this approach enables the prediction network to learn efficiently and maintain a consistent performance even in different road segments. The details of this problem are discussed in Section 3.1.
- We have proposed a road-aware, sequence-to-sequence trajectory prediction network. Using the fact that a vehicle naturally runs along the shape of the road, we have developed an output constrained prediction network. By combining a deep learning network with the prior knowledge of the roadway, the structural limitations of the roadway have been incorporated in the network. Consequently, the proposed trajectory prediction network is able to predict a feasible and realistic intention of a driver and trajectory of the surrounding vehicles.

The remainder of this paper is organized as follows: a description of previous studies is presented in Section 2. The methodology developed in this work according to the primarily investigated problem statement is described in Section 3. Details of the experimental results, which prove the superior performance of the proposed method, are presented in Section 4. Conclusions from the present work along with aspects of future work are given in Section 5.

## 2. Related Work

Before learning-based approaches were applied, the commonly used classical method for predicting the state of a vehicle was KF. This method exhibits good performance in the application of on-road tracking and short-term prediction [5,6]. However, it has limitations in long-term predictions since it does not consider the future transitions of a driver’s intention. To improve the long-term accuracy of KF, it was combined with maneuver recognition techniques such as the hidden Markov model (HMM) [16] and the dynamic Bayesian network (DBN) [17]. However, it is difficult to derive all models of KF corresponding to the different maneuvers. Meanwhile, due to the excellent performance of deep-learning-based approaches, significant improvements in trajectory prediction were made. The long short-term memory (LSTM) encoder–decoder structure [8] with an occupancy grid map was first proposed for probabilistic trajectory prediction on highways. In addition, a few studies [18,19] showed that trajectory prediction can be improved by handling multi-modal uncertainty with maneuver recognition such as lane change left (LCL), lane change right (LCR), and lane keeping (LK). Subsequently, various deep learning structures have been proposed by combining the LSTM encoder–decoder with conditional VAE [10,11,12] for generating diverse trajectories. These studies focus mainly on the deep learning architecture to improve the prediction accuracy and to solve the uncertain nature of the trajectory prediction problem. However, in such studies there is a possibility of the method predicting an infeasible trajectory, since they do not consider road structure, and a practical use of road information needs to be considered to learn a real dataset efficiently.

On the contrary, the use of road information in trajectory prediction has been known to be significantly beneficial [14,15,17]. There have also been attempts to include road information in applications employing deep learning techniques. A few studies [20,21] have proposed rasterizing a vector layer of road information into an RGB space in order to incorporate the road features in the prediction network. However, handling a road environment as a rasterized image is computationally taxing, since it requires an additional convolutional neural network (CNN). In contrast to the above-mentioned studies, our methodology extracts useful information in advance from the HD maps and thus naturally reduces the network complexity and computational burden.

There are other related works that consider interactions between vehicles. However, our study does not include these interactions for the following reasons: firstly, on a highway, the traffic runs almost parallel, which causes sensor occlusion, and secondly, longitudinal inter-distance between vehicles is relatively larger than in an urban environment. Therefore, it is realistically difficult to apply the interactions between vehicles to the prediction algorithm while applying it to highway traffic. Although several studies have considered these interactions, these works have been performed at an uncontrolled intersection [11,21] where an interaction could be easily captured or evaluated [22,23] on the next-generation simulation (NGSIM) dataset [24]. The highway traffic in these cases has been captured using a camera mounted on top of a building. In addition, these studies implicitly assume that information on the state of a vehicle can be obtained regardless of the range and location of the mounted sensors.

## 3. Problem Statement and Methodology

In this section, we describe the problem statement and propose the methodology to solve this problem.

### 3.1. Practical Problem Statement of Trajectory Prediction

**Figure 2:**
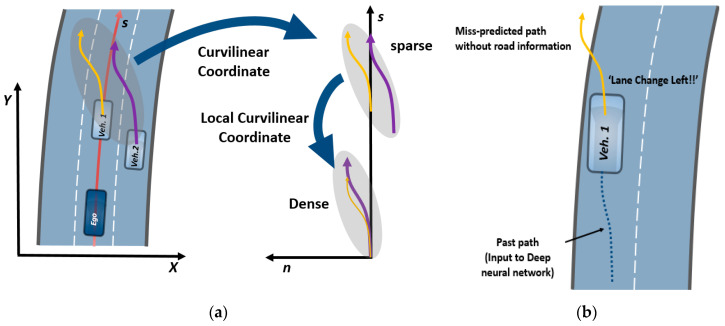
Practical problem statement. (**a**) If we represent trajectories of the surrounding vehicles in the local curvilinear coordinate system, the data distribution becomes compact; (**b**) Wrong prediction is caused by a zigzag motion of the vehicle without any road constraint.

It is inevitable to collect a dataset by oneself for developing a deep-learning-based trajectory prediction algorithm. However, one has to face certain practical problems in this regard. Firstly, a dataset that covers the complete range of the sensor cannot be collected. For example, even if two vehicles shown in Figure 2a change lanes in an almost identical manner, they are perceived differently in the deep learning network because the measured position values of each vehicle are distributed differently. In short, this implies that we must collect a dataset which contains every maneuver that could happen within the entire range of the sensor. However, this requires an enormous amount of manpower and time. Secondly, it is realistically difficult to collect lane change (LC) trajectories because drivers do not frequently change their sane while driving on a highway. Therefore, a method of using the sparsely collected dataset appropriately should be considered. A graphical description and the expected result of such a dataset is shown in Figure 2a. Further, there could be a zigzagging motion of a vehicle in a real traffic situation, and this can sometimes cause the maneuver recognition network (MRN) to output an infeasible maneuver. However, an intelligent vehicle should be robust to such unlikely maneuvers of surrounding vehicles. Figure 2b shows a typical example. To mitigate these problems, we adopted the curvilinear coordinate system and lane assignment to represent the motion of a vehicle using HD maps. Consequently, this enables data-efficient learning and prediction of feasible future trajectories.

### 3.2. Extracted Road Information from HD Maps

In Section 2, we mentioned that the performance of trajectory prediction can be improved by using road information. In this section, we describe the steps adopted in this work for extracting road information from HD maps and explain how these can be utilized for trajectory prediction.

**Figure 3:**
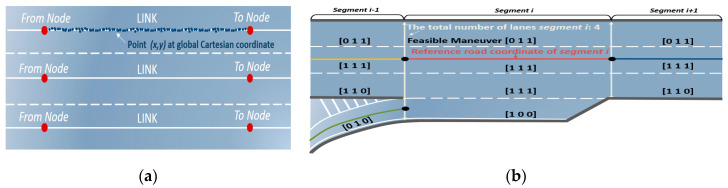
Extraction of road information. (**a**) HD maps contain nodes and links and each component is represented by points in the Cartesian coordinate system; (**b**) After extracting information via offline preprocessing, each segment has information corresponding to the segments such as B-spline parameters, lane information, length of the segment, and a feasible maneuver one-hot vector.

HD maps usually include a point cloud for recording data from the surrounding environment of a roadway and the position data of the probed vehicle, collected using a high-resolution light detection and ranging (LIDAR) instrument and a high-precision global navigation satellite system/inertial navigation system (GNSS-INS), respectively. The data are usually provided as a database. An example of the data is shown in Figure 3a in which the highway road information is divided into segments and each segment includes a line connecting the start and end points. Each line is called a link and each point is called a node. Using this provided database, we extracted the following information via offline processing:

- Reference curvilinear coordinates: B-spline parameters approximating the corresponding line;
- Additional information about the segment: the total number of lanes, the reference lane, and the length of the segment;
- Feasible maneuver vector for each lane: the simplest way to generate a feasible maneuver vector is the one-hot vector representing the possible maneuvers among LCL, LK, and LCR. For example, in the case of the first lane in Figure 3b, it is [0 1 1] because a vehicle can only execute LK and LCR owing to the road shape. However, it should be noted that any real values between 0 and 1 can be inputted.

### 3.3. Data Processing

**Figure 4:**
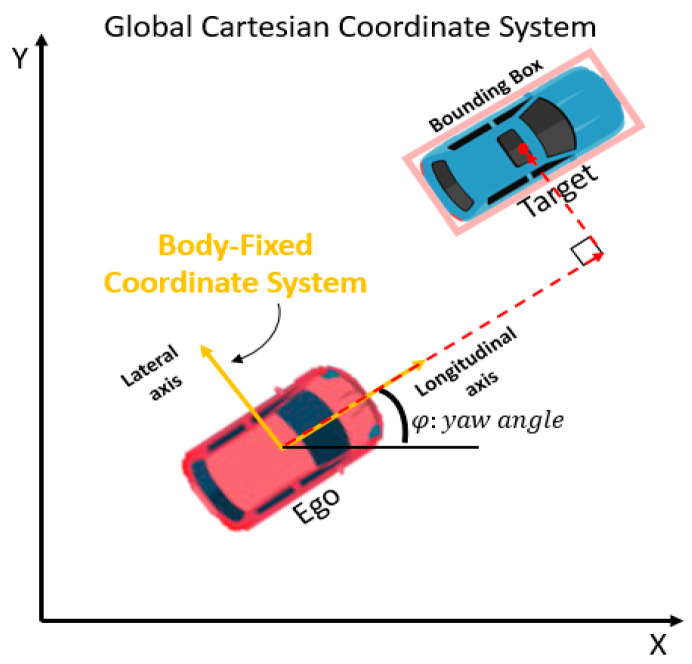
Global Cartesian coordinate system and body-fixed coordinate system for the representation of vehicle states.

From this data processing, we assume that one can obtain adequately accurate tracking information, and the necessary vehicle state tracking information are presented as follows (see Figure 4).

- Ego-vehicle states: global position, yaw angle, longitudinal velocity and lateral velocity;
- Target vehicle states: longitudinal relative displacement, lateral relative displacement, longitudinal relative velocity and lateral relative velocity of centroid of 2D bounding box.

In fact, the performance of the trajectory prediction is affected by sensor accuracy and tracking algorithm. To obtain accurate tracking information, therefore, we used commercial sensors such as high-precision GNSS-INS integrated system which ensures global position error within 1 cm and an LIDAR sensor which ensure relative state tracking error of the target vehicle within 10 cm.

**Figure 5:**
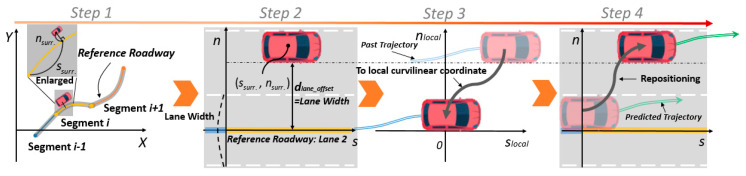
Description of the data processing steps. Step 1: Global Cartesian coordinates; Step 2: Transformation to the curvilinear coordinates; Step 3: Translation to the local curvilinear coordinates; Step 4: Repositioning to the curvilinear coordinates.

Data processing consists of four steps, which have been graphically represented in Figure 5, as follows:

1. Step 1: We assume the global position of an ego vehicle to be $\left(X_{ego},Y_{ego}\right)$ and obtain the relative states of the surrounding vehicles using GNSS-INS and LIDAR, respectively. Then, we obtain the global position $\left(X_{surr.},Y_{surr.}\right)$ of the surrounding vehicles. Next, we find the corresponding segment on which the surrounding vehicle is located by using the position values of the “From” and the “To” nodes. Here, we can search for it first within a range which spans from the back to the front of the segment of the ego vehicle;
2. Step 2: Once the segment is found, it indicates that the B-spline parameters, which approximate the reference roadway, have been obtained. Then, we can find the point $\left(s_{surr.},n_{surr.}\right)$ which is the orthogonal and the nearest point to the segment (refer to the enlarged drawing in Step 1 of Figure 5.) The detailed algorithm to find this point is omitted here, since it not part of our study’s contribution. However, readers can refer to Ref. [25] for further details. After obtaining the curvilinear coordinates, we can assign the lane number to which the surrounding vehicle belongs to. Lane assignment does not require real-time determination, however, it is sufficient to use a simple threshold and a count algorithm with lateral displacement $n_{surr.}$ in this step. The converted curvilinear coordinates are stored in chronological order;
3. Step 3: All the sequential coordinates are transformed to the local curvilinear coordinate system in which the curvilinear coordinates are translated by $\left(s_{surr.},\;n_{lane\_offset}\right)$. In this way, the trajectories of all the surrounding vehicles are represented on the same frame of reference even though their positions are different. We define this as the local curvilinear coordinates. After transforming the position values, the velocities $\left({\dot{s}}_{surr.},\;{\dot{n}}_{surr.}\;\right)$ in the local curvilinear coordinates are obtained by simply projecting the velocities in the global Cartesian coordinates to the local curvilinear coordinates. In this way, we can generate a compact dataset by carrying out the above-mentioned procedure. Subsequently, the transformed sequential trajectory points are directly fed to the trajectory prediction network as an input sequence. The detailed trajectory prediction network architecture is described in Section 3.4;
4. Step 4: Step 4 follows the trajectory prediction network. Here, we simply reposition the predicted trajectory to the curvilinear coordinate system. Following this, depending on the user’s purpose, such as a collision assessment or trajectory planning, the predicted trajectory can be used as is or translated to the Cartesian coordinates.

**Figure 6:**
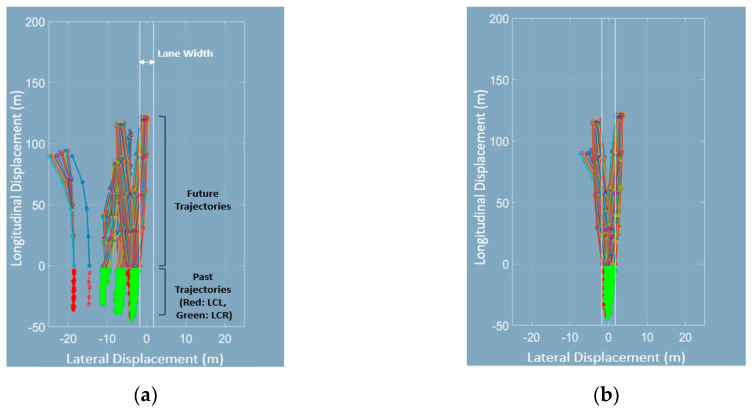
Results obtained after data processing and comparison of trajectories before and after lane assignment. For better visualization, in other words, we intend that one is not translated laterally, and another one is completely translated to the local curvilinear coordinate. (**a**) shows the trajectories transformed to the local curvilinear coordinates without lane assignment, whereas (**b**) shows the trajectories transformed to the local curvilinear coordinates with lane assignment.

Brief results and the effectiveness of data processing are shown in Figure 6 below. We chose and visualized 200 arbitrary trajectories of lane change data. Figure 6a,b shows the results processed by Step 3 without lane assignment and Step 3 with lane assignment (i.e., in the local curvilinear coordinates), respectively. As can be seen from the figures, the data distribution in Figure 6b is compact. In addition, all the lateral displacements of the data in Figure 6a have negative values. This indicates that the trajectory prediction may fail if lane change occurs in the positive domain. On the contrary, Figure 6b shows concise lane change trajectories because all data are represented in the local curvilinear coordinates. With these data, the trajectory prediction network is able to learn the data more efficiently.

### 3.4. Road-aware Trajectory Prediction Network

In this work, the ultimate goal is to predict the long-term future trajectory $\tilde{y}=\left[{\tilde{y}}_{t+1},{\tilde{y}}_{t+2},\;\ldots ,\;{\tilde{y}}_{t+T_{p}}\right]$ for a given past trajectory $\tilde{x}=\left[{\tilde{x}}_{t-T_{b}-1},\;{\tilde{x}}_{t-T_{b}},\;\ldots ,\;{\tilde{x}}_{t-1}\right]$ and maneuver constraints $C=\left[c_{1},\;c_{2},\;c_{3}\right]$. Here, ${\tilde{y}}_{i}$ and ${\tilde{x}}_{i}$ are defined as ${\tilde{y}}_{i}=\left[s_{i},n_{i}\right]$ and ${\tilde{x}}_{i}=\left[s_{i},n_{i},{\dot{s}}_{i},\;{\dot{n}}_{i}\right],$ and the components of maneuver constraints vector C(see Figure 3b) are real values between 0 and 1. Note that we do not include any feature to take interactions into account, as explained in Section 2. The trajectory prediction module must be able to handle uncertainties, i.e., multiple futures. To this end, we adopted a multi-maneuver and VAE-based encoder–decoder architecture [10,12]. The proposed architecture consists of maneuver recognition and trajectory regression parts. Herein, maneuver recognition and trajectory regression account for uncertain driver maneuver and sampling diverse learned trajectories corresponding to each maneuver, respectively. Many researchers have proposed architectures similar to ours [18,22]. However, our MRN considers the structural limitations of the road as constraints with a constrained loss function. As a result, this naturally keeps the prediction module from predicting an infeasible trajectory. It should be noted here that one could exploit other sequence-to-sequence network variants since our data processing described in Section 3.3 simply modifies the input data to maximize the efficiency of learning. We present a summary of the architecture details in Table 1. In our architecture, to encode past trajectory and future trajectory, a gated recurrent unit (GRU) [26], which is one of the RNN variants, was used, since this has a simpler structure than LSTM. Therefore, GRU takes less time to train and is more efficient. VAE consisted of only fully connected layers. Column 4 and 6 in Table 1 present the input and output size of each layer, and the dimensionality was adjusted to match the input and the output of the front and rear layer. Lastly, a rectified linear unit (ReLU) [27] was selected as the activation function to take account for nonlinearity.

#### 3.4.1. Maneuver Recognition Network

Our MRN was motivated by a weakly supervised learning used for medical image analysis. H. Kervadec et al. [28] imposed inequality constraints for a weakly supervised semantic segmentation of medical images. They proposed a differentiable loss function which handles inequality constraints and accommodates standard stochastic gradient descent. Adopting this loss function, we realized a constrained MRN. MRN consists of GRU-encoder2 and FCL2. GRU-encoder2 encodes past trajectory of the target vehicle, and then the output of GRU-encoder is concatenated with prior knowledge, that is, the structural constraints vector of the road, which we extracted via offline processing in advance as mentioned in Section 3.2. Subsequently, this concatenated vector passed through the FCL2 and softmax function, and finally we could obtain the output which is the estimated probability corresponding to each maneuver. To realize constrained MRN, we finally constructed the constrained loss as follows

(1) $$Loss_{MRN}=\overset{N}{\underset{i=1}{\sum }}\overset{3}{\underset{m=1}{\sum }}p_{m,i}log\left({\hat{p}}_{m,i}\right)+\left(1-p_{m,i}\right)log\left(1-{\hat{p}}_{m,i}\right)+\lambda_{constraint}B\left({\hat{p}}_{m,i}\right),$$

where $p_{m,i}$ and ${\hat{p}}_{m,i}$ are the ground truth (GT) of the maneuver and the output of the MRN, respectively, N is batch size, $\lambda_{constraint}$ is the weight parameter corresponding to the constraint, and the function $B\left({\hat{p}}_{m,i}\right)$ is given by

(2) $$B\left({\hat{y}}_{m,i}\right)=\{\begin{array}{cc} {\left({\hat{p}}_{m,i}-c_{m,i}\right)}^{2}, & \mathrm{if}\;p_{m,i}\geq c_{m,i}\\ 0, & \mathrm{otherwise} \end{array}.$$

here, $c_{m,i}$ is the value of the aforementioned inequality constraint vector. By introducing the function $B\left({\hat{p}}_{m,i}\right)$, $Loss_{MRN}$ acts as a barrier to prevent an infeasible maneuver. The loss function in Equation (1) is nothing but a typical cross-entropy loss function with a barrier function B. This acts like the cross-entropy loss without the barrier function, but the barrier function would be active if the output of MRN violated inequality constraints. For example, [1 1 0] indicates that the LCL and LK of maneuvers could only happen according to the structure of road. Therefore, each value of the one-hot vector is imposed to a loss function as an inequality constraint.

#### 3.4.2. Trajectory Prediction Network

**Figure 7:**
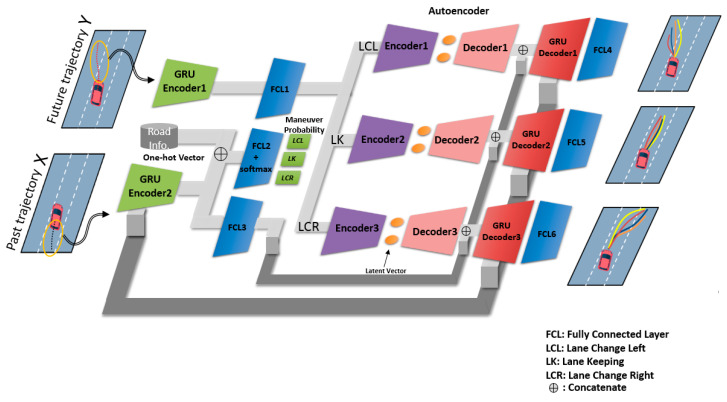
Schematic of the trajectory prediction network.

The trajectory prediction must have the ability to generate diverse trajectory samples, since different drivers have different driving characteristics, even if the driving maneuver is the same. Similar to other studies, our trajectory prediction network also adopts the generic encoder–decoder architecture with a VAE, but a slightly different structure. We have divided the prediction part into three VAE-GRU decoder networks to sample the trajectories which correspond to each maneuver (see the final outputs in Figure 7). GRU-encoder1 encodes the future trajectory, while VAE-encoders are learned to model an ideal distribution *z*, called a latent variable, to encode the future trajectory. The decoder generates diverse trajectories combined with the GRU-decoder, where its hidden states are connected to hidden states of the GRU-encoder2. Here, the ideal distribution *z* is modeled as a two-dimensional Gaussian distribution (i.e., *z* ~ $N(\mu_{z},\sigma_{z})$). Accordingly, in the inference phase, we remove the encoder parts of the VAE and generate a diverse future trajectory by sampling the variable *z* from the learned distribution. This sampled *z* passes through a decoder, and subsequently, the output of the decoder is concatenated with the encoded input feature. Finally, after passing through the last fully connected layer, we obtain the various trajectory samples (see the final output in Figure 7). Note that even though each of the encoder–decoder networks have the same structure, they do not share their parameters. To optimize this network, the trajectory prediction loss function is defined as follows

(3) $$Loss_{path}=\overset{3}{\underset{m=1}{\sum }}I_{m}\left[\frac{1}{T_{p}}\overset{T_{p}}{\underset{t=1}{\sum }}\|y_{t,m}-{\hat{y}}_{t,m}\|{}_{2}+\beta \cdot KLD\left[Q_{\emptyset }\left(z_{m}|Y\right)\right]\right],$$

where $I_{m}$ is a binary indicator value, which is equal to 1 for the GT of the maneuver and 0 otherwise, and KLD is the Kuller–Leibler divergence [29]. The first term on the right-hand side is the trajectory regression loss and the second term makes the learned Gaussian model close to a Normal distribution. This loss function is enabled to only update parameters of the corresponding network by a binary indicator value, and we have also used the re-parameterization technique [30] to sample *z,* since it is impossible to sample during backpropagation.

## 4. Experimental Results and Analysis

### 4.1. Dataset

**Figure 8:**
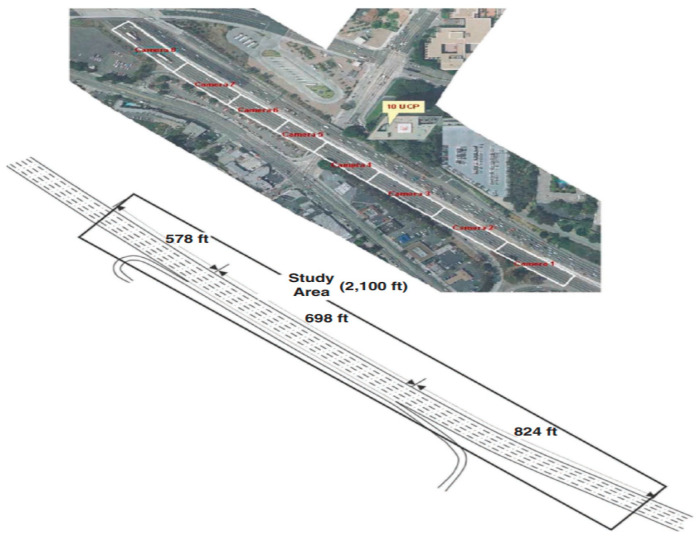
Image from the US highway 101 dataset [31].

For large-scale evaluation and a proof concept, we chose a publicly available I-101 [24] dataset which consists of images of the United States Interstate-101 freeway traffic captured by a camera, mounted on top of a building, at a frequency of 10 Hz (see Figure 8). These images include a large amount of real traffic trajectories as well as merging sections which are especially beneficial for validating the feasibility of the predicted trajectories in this study. We extracted 199,477 trajectories that consist of 170,494 LK and 28,983 LC trajectories, respectively.

### 4.2. Results and Analysis

#### 4.2.1. Effectiveness of The Local Curvilinear Coordinates

**Figure A1:**
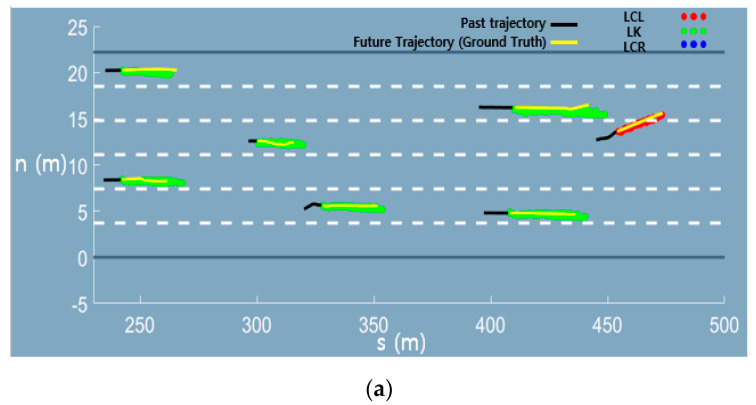

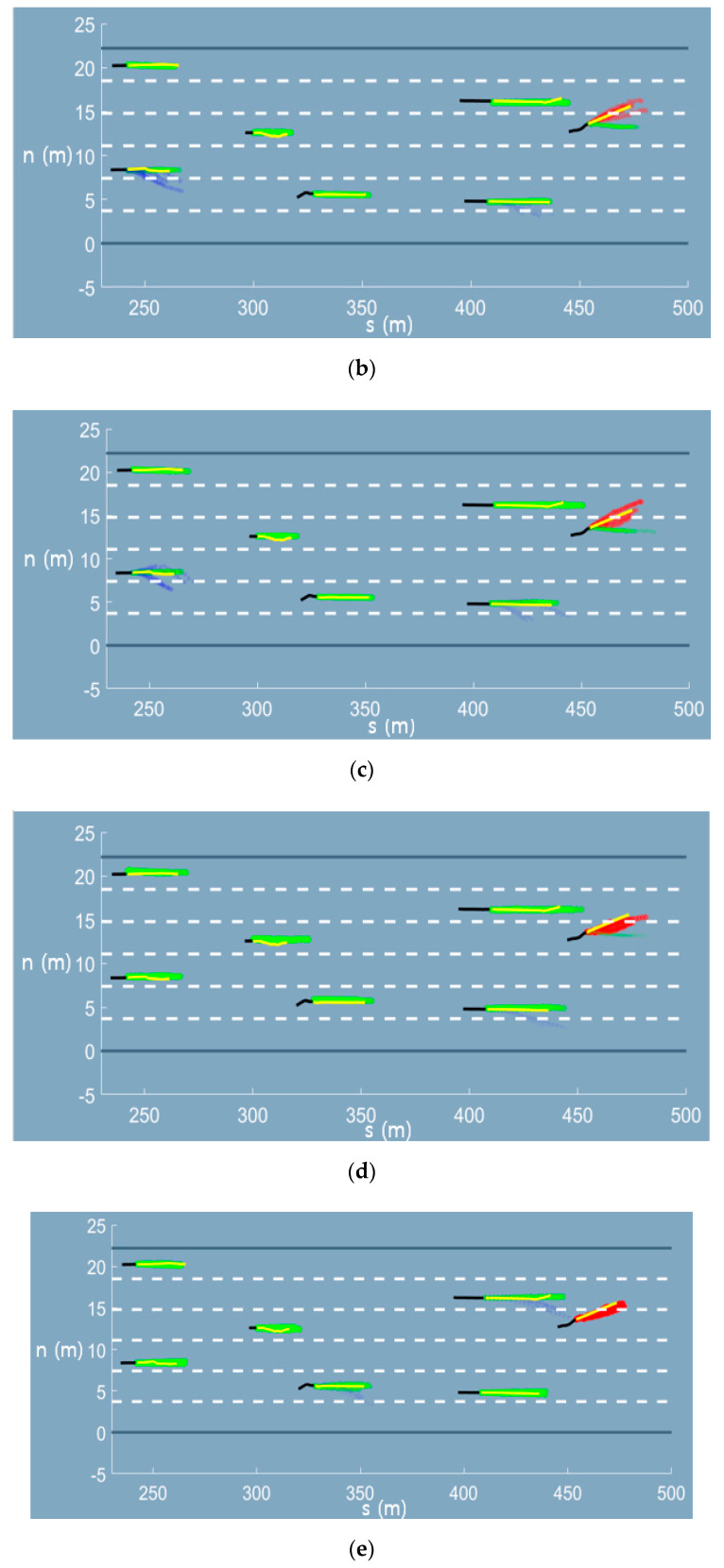

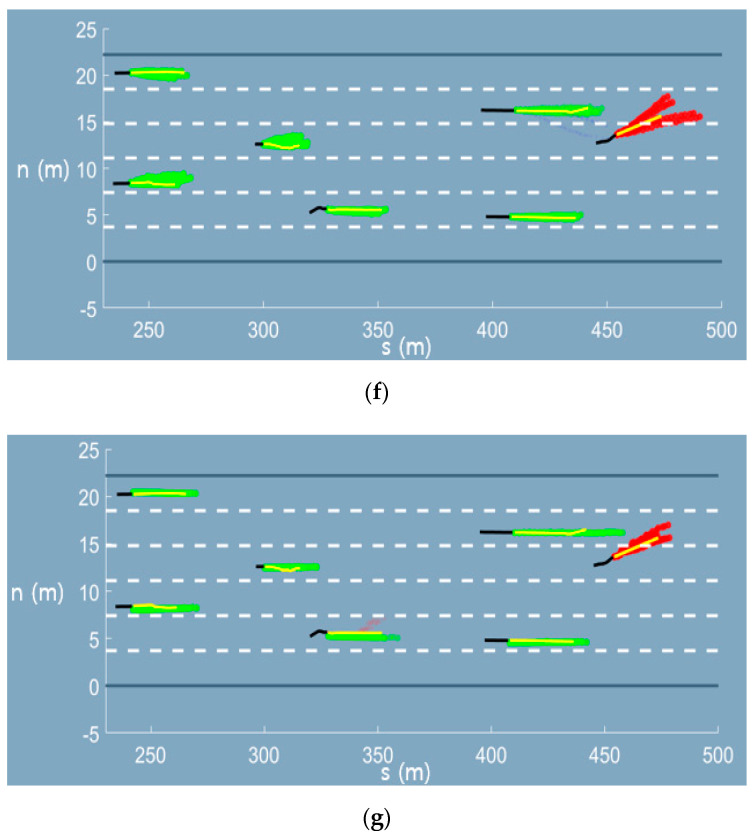
Results of the trajectory prediction network in the local curvilinear coordinates. (**a**–**g**) are the results of learning by using 5%, 10%, 15%, 20%, 40%, 60%, and 80%, respectively, of the dataset. The bigger and more apparent the marker, the higher the confidence level of the maneuver.

We mentioned in Section 3.1 that the trajectory prediction network can learn data efficiently when the trajectories are represented in the local curvilinear coordinate system. In other words, because the data are compactly arranged using the proposed method (see Figure 6), it requires less data and less network capacity for predictions. To verify this aforementioned effectiveness, we split the entire dataset from 5% to 80%, and the remainder 20% was used as a test dataset. We represented each dataset using the two different coordinate systems, processed by Steps 2 and 3, respectively, of the data processing described in Section 3.2. For conducting a fair comparison, we set all the parameters and the network structure to be equal and carried out the test by gradually increasing the amount of data. The quantitative test results are presented in Table 2, and results are presented graphically in Figure A1 of Appendix A for better visibility.

The evaluation metric used in this case is the weighted minimum average displacement error (WMADE) [11,32,33], which is the weighted sum of the predicted trajectories, corresponding to each maneuver, that are closest to the GT trajectory. This metric is well known for multiple future prediction tasks, and it is expressed by the following equation

(4) $$WMADE=\frac{1}{N}\overset{3}{\underset{m=1}{\sum }}w_{m}\underset{k\in K}{\min}\frac{1}{T_{p}}\overset{T_{p}}{\underset{t=1}{\sum }}\|y_{t,m}-{\hat{y}}_{t,m}\|,$$

where the weights $w_{m}$ are outputs of the MRN and *K* is the sample number, set to 5.

**Figure A2:**
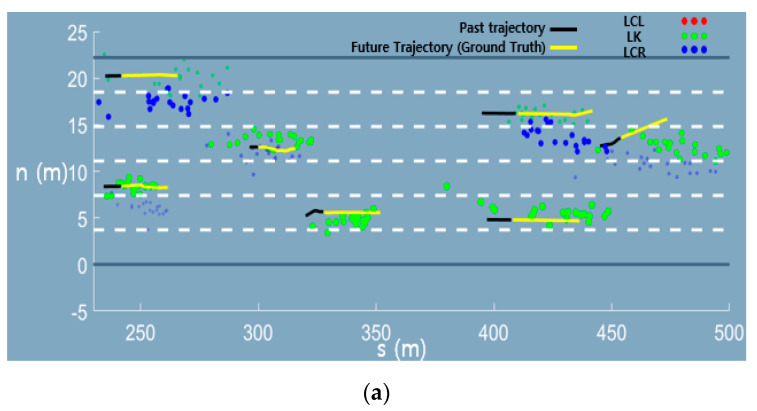

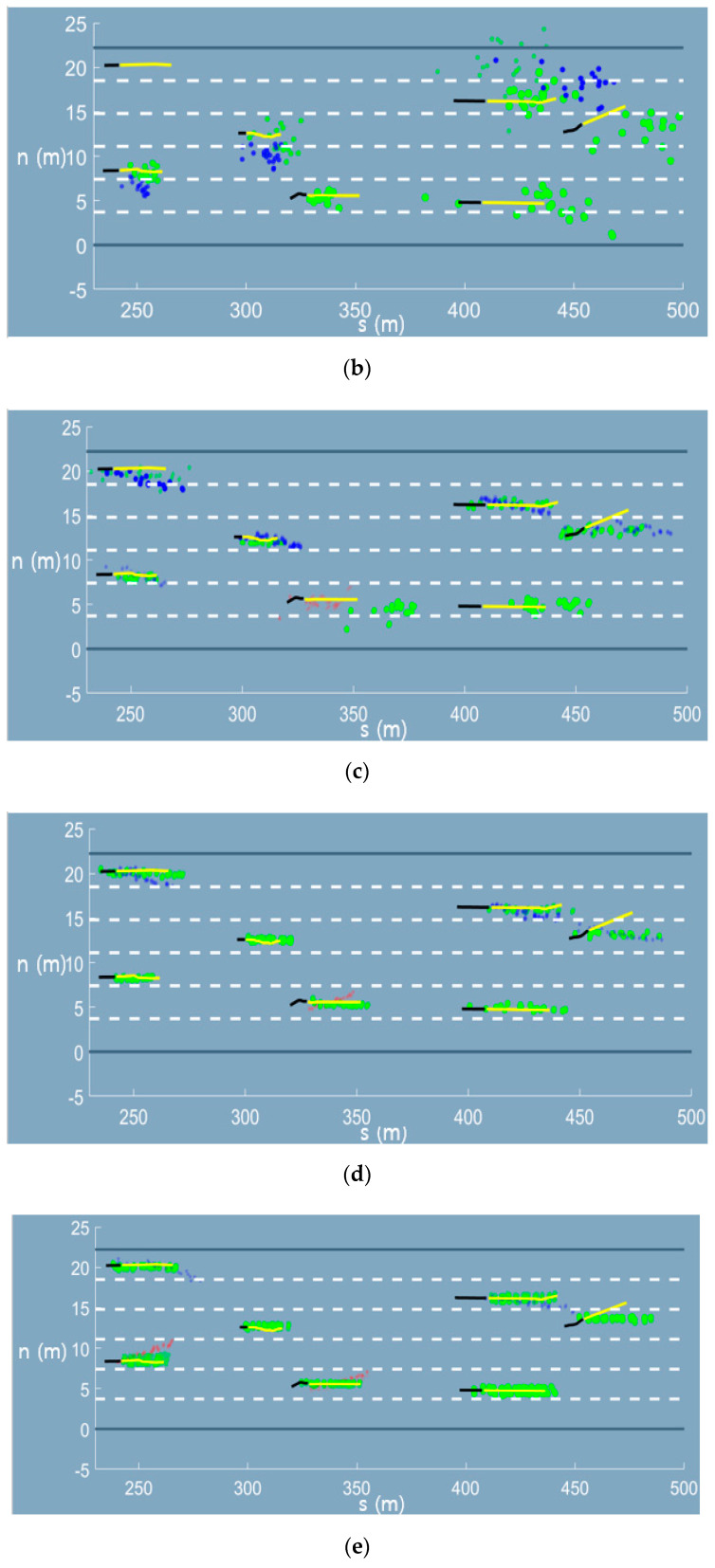

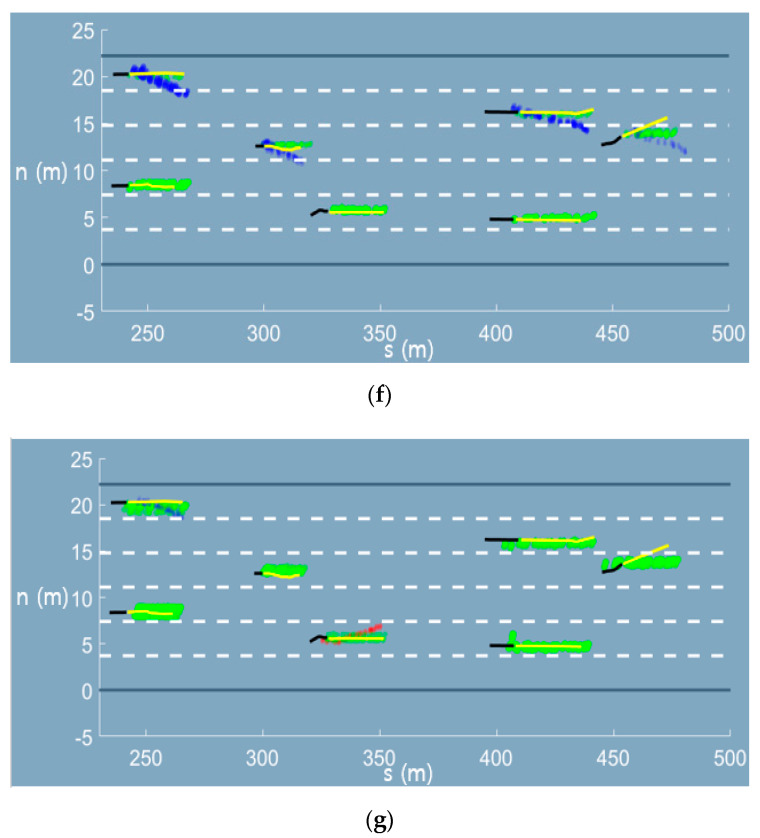
Comparative results of the trajectory prediction network. (**a**–**g**) are the results obtained by learning from 5%, 10%, 15%, 20%, 40%, 60%, and 80%, respectively, of the training dataset. The bigger and more apparent the marker, the higher the confidence level of the maneuver.

The WMADE values for the total horizon are given in column 2 of Table 3. From Table 2, it can be seen that for the proposed method, WMADE starts from 1.53 when 5% of the learning dataset is used and ends with 1.13 when 80% of the learning dataset is used. Furthermore, the value seems to be saturated for 60% dataset usage. On the contrary, the prediction for the curvilinear coordinates starts from 9.52 at 5% of dataset usage and ends with 3.30 at 80% of dataset usage. The results for 4 s are especially remarkable. The difference between the WMADE values at 5% and at 80% of dataset usage for the proposed method is only 0.99, whereas the difference for the curvilinear coordinates is 8.06. This indicates that the coordinate transformation to the local curvilinear coordinates is reasonable and efficient for representing the trajectories of an on-road vehicle. Furthermore, Figure A1 and Figure A2 in Appendix A show the graphical results of the proposed method and the comparative method, respectively. The difference between these results is quite clear. For our method, although the learning result corresponding to 5% of the dataset looks deterministic as only 5% of the dataset does not contain diverse trajectories, its prediction samples are not too far from the GT. As the amount of the learning dataset increases, the diversity of the predicted trajectory samples corresponding to each maneuver evidently improves. On the contrary, it is difficult to judge whether the results obtained from the comparative method using only 5–15% of the learning dataset have been learned sufficiently, since the shape of the predicted trajectory samples is not yet complete. The prediction result gradually converges to the motion of a car, but the distribution of datasets is too wide to sample plausible trajectories. Thus, we conclude that our method provides data-efficient learning in situations where fewer datasets are available. This is an expected result because the road geometry of each lane is very similar to each other, although the trajectory values themselves could be different.

Now we compare our result to other related works. In this comparison, we report root mean square error (RMSE) in order to utilize values in their works.

- NLS-LSTM [34]: This model is an encoder–decoder architecture that has local and non-local operation to capture interaction;
- M-LSTM [18]: This model is an encoder–decoder architecture that considers adjacent six vehicles of target vehicle and encode vehicle’s maneuver. This maneuver encoding vector is used to predict multi-modal trajectory prediction;
- MATF [35]: This model is an encoder–decoder architecture that uses multi-agent tensor to capture interaction.

It should be noted here that all of the other works consider interaction between vehicles, while our method does not, as mentioned in Section 2.

Although the proposed method is in the third place for 1 s and 2 s, the longer prediction horizon the better our result is. Specifically, our result outperforms the other for 4 s. This suggests that making a compact distribution of datasets through the proposed data processing has an advantage in the form of a longer prediction horizon. Normally, though uncertainty increases for the longer prediction horizon due to various and different driver characteristics, a unified representation of the local curvilinear coordinate helps to improve learning efficiency. However, our methodology is not just better, but the comparative methods can be improved by our data processing method and vice versa. In fact, our proposed data processing can be applied to other sequence-to-sequence architecture. For example, the local coordinate representation is combined with features to capture interaction, and then this is injected to their prediction network as an input.

#### 4.2.2. Feasible Trajectory Prediction at a Merging Section

**Figure 9:**
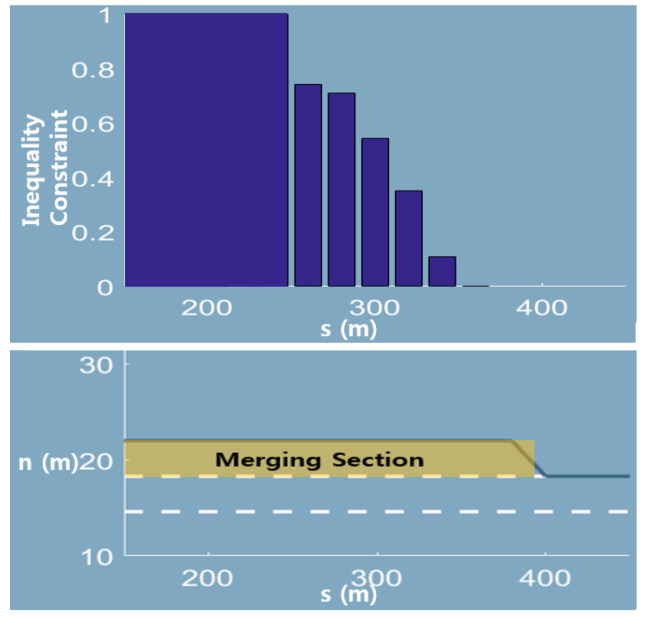
Merging section of I-101 and inequality constraints. The upper plot presents the inequality constraints setting as a function of the distance till the end of the merging section, whereas the lower figure gives a graphical description of the merging section.

Merging sections at freeways is especially useful for evaluating the effectiveness of the inequality constraint. Because cars cannot execute LK as they approach the end of the merging section, this fact naturally leads to the constraint of the LK maneuver, i.e., the maximum probability of LK. In this work, we extracted trajectories at a merging section of NGSIM I-101. The statistical ratio of LK execution by distance is shown in Figure 9. The statistical ratio by distance was set to the inequality constraint.

It should be noted here that the constraint is discrete and is a user-defined function by prior knowledge. Therefore, it could be any function of distance till the end of the merging section, such as a half-Gaussian-like function. For performing a quantitative evaluation of the effectiveness of the constraint, we chose the Top1 ADE. This parameter is an average displacement error of the maneuver that has the highest probability of MRN output. In fact, the most likely maneuver of the surrounding vehicles is critical for motion planning, since their decision making is affected the most by this maneuver.

**Figure 10:**
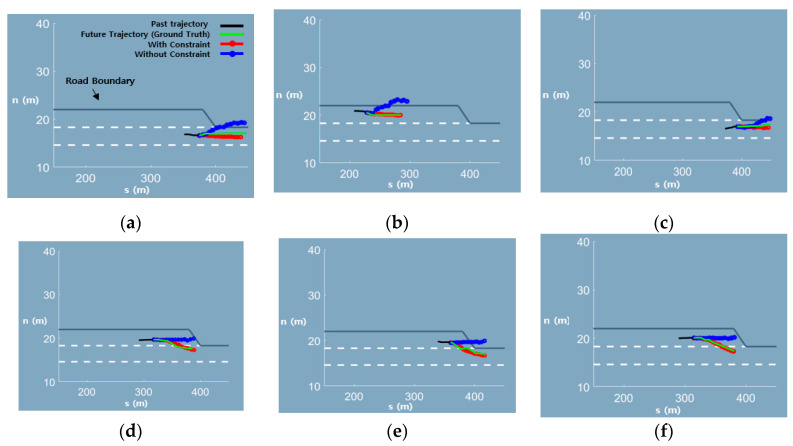
Comparison of the trajectories predicted by the trajectory prediction network with (red line) and without (blue line) constraint. (**a**–**c**) show that the trajectory prediction network without constraint predict LCL as the most probable maneuver despite the road boundary on the left side of the car. (**d**–**f**) show that the prediction network without constraint predict LK as the most probable maneuver despite its proximity to the end of merging section while the trajectory prediction network with constraint predict LCR.

To conduct a fair evaluation, we equalized all network structures and learning parameters. However, in one case, the constrained loss has been included in the MRN, whereas in the other case, a typical cross-entropy loss was included in MRN. In this test, we set the sample number to 1 for better visibility of results, as shown in Figure 10.

As shown in Table 4, the prediction result with the constraint is superior to that without any constraint. This indicates that it is reasonable to consider the road structure as a constraint because vehicles are affected by the road structure. Moreover, Figure 10 shows a representative critical scene. The result without the constraint corresponds to a realistically infeasible prediction. Nevertheless, vehicles cannot run outside the roadway. The network predicts that the vehicle is going through a road boundary. In addition, when the vehicle approaches the end of the merging section, it must merge into the freeway in the near future by changing lanes, even though vehicles seem to maintain their lanes. However, the network without a constraint cannot reflect this fact. In this case, the prediction result could cause a severe accident. On the contrary, the network with a constraint is aware of the road structure and thus can predict a reasonable trajectory. The networks never predict LCL as the most likely maneuver and predict LCR in advance at the end of the merging section.

## 5. Conclusions and Future Work

In this work, we proposed a trajectory prediction network which especially maximizes the use of road information. For data-efficient learning, we used the local curvilinear coordinate system to represent the trajectories compactly using B-spline interpolation and lane assignment. The results have been evaluated by increasing the amount of data, and for the first 5% of the dataset, our proposed network shows a superior performance. In particular, the longer the prediction horizon, the more remarkable was the difference observed. Thus, it can be concluded that a trajectory prediction network that learns small amounts of data results in a prediction accuracy is comparable to the results obtained using large amounts of data. In addition, we included inequality constraints in the trajectory prediction network. Without the additional feature extraction network of the roadway, our trajectory prediction network can be compliant with a road structure and shows superior performance especially in the merging section. Moreover, even though the vehicle tends towards remaining in the LK mode, the proposed MRN predicts LC at the end of the merging section. This helps in feasible trajectory prediction as well as in making safe decisions.

On the other hand, our work has limitations that should be further discussed. First, various critical experiments must be carried out for real application. An output of the prediction task should be fed to a decision-making module. Therefore, we have to verify online inference performance with a decision-making module in various cases. Additionally, for trajectory prediction tasks in an urban environment, some works must be extended. For example, a unified representation of the complex urban road network needs to be devised, and our network needs to be extended to cover a variety of executable maneuvers such as U-turns, left turns, and right turns. Furthermore, inter-vehicle interactions must be considered for an accurate future trajectory prediction.

## Figures and Tables

**Table 1:** Details of the Road-Aware Trajectory Prediction Network.

Part	Name		Layer Type (Depth)	Input Size	Activation	Output Size
MRN (Classification)	GRU-encoder1		GRU (2)	4	ReLU	32
	FCL2		Fully Connected (2)	35 16	ReLU ReLU	16 3
Trajectory (Regression)	GRU-encoder2		Same as GRU-encoder1			
	FCL1,3		Fully Connected (1)	32	ReLU	16
	VAE	VAE-encoder1,2,3,	Fully Connected (2)	16 8	ReLU ReLU	8 2
		VAE-decoder4,5,6	Fully Connected (2)	2 8	ReLU ReLU	8 16
	GRU-decoder		GRU (2)	32	ReLU	16
	FCL4,5,6		Fully Connected (2)	16 8	ReLU ReLU	8 2

**Table 2:** Comparison of the weighted average displacement error (ADE) values between the proposed local curvilinear coordinates and the curvilinear coordinates.

Weighted ADE (m) (Local Curvilinear Coordinate/Curvilinear Coordinate)					
Percentage (%)	Total	1 s	2 s	3 s	4 s
5	1.53/9.52	0.61/8.34	1.39/8.05	2.29/9.44	3.21/12.29
10	1.26/7.26	0.57/5.72	1.20/6.57	1.80/8.06	2.43/10.54
15	1.24/5.49	0.58/4.22	1.18/4.73	1.75/6.15	2.40/7.39
20	1.17/5.25	0.49/4.70	1.10/4.33	1.68/5.45	2.36/6.69
40	1.15/3.56	0.49/3.07	1.11/3.18	1.64/3.85	2.28/4.67
60	1.14/3.70	0.52/3.24	1.08/3.28	1.64/3.81	2.23/4.68
80	1.13/3.30	0.49/3.00	1.05/2.90	1.63/3.41	2.22/4.23

**Table 3:** Comparison of root mean square error (RMSE) values between the proposed method and comparative methods. The best is marked in bold and the second best is underlined.

RMSE (m)				
Method	1 s	2 s	3 s	4 s
M-LSTM	0.58	**1.26**	2.12	3.24
MATF	0.67	1.51	2.51	3.71
MLS-LSTM	**0.56**	1.22	**2.02**	3.03
Ours	0.65	1.36	2.12	**2.94**

**Table 4:** Comparison of the Top1 ADE values corresponding to the prediction with and without constraints at a merging section.

Top1 ADE (m)					
	Total	1.0 s	2.0 s	3.0 s	4.0 s
w/constraint	1.09	0.49	0.98	1.60	2.44
w/o constraint	1.20	0.52	1.07	1.77	2.69
